# Transcriptomic analysis reveals gender differences in gene expression profiling of the hypothalamus of rhesus macaque with aging

**DOI:** 10.18632/aging.103682

**Published:** 2020-09-27

**Authors:** Yong Fan, Congru Li, Wendi Pei, Tao Tan, Rong Li, Jie Qiao, Yang Yu

**Affiliations:** 1 Key Laboratory for Major Obstetric Diseases of Guangdong Province, Key Laboratory of Reproduction and Genetics of Guangdong Higher Education Institutes, The Third Affiliated Hospital of Guangzhou Medical University, Guangzhou 510150, China; 2 Center for Reproductive Medicine, Department of Obstetrics and Gynecology, Beijing Key Laboratory of Reproductive Endocrinology and Assisted Reproductive Technology and Key Laboratory of Assisted Reproduction, Ministry of Education, Peking University Third Hospital, Beijing 100191, China; 3 Yunnan Key Laboratory of Primate Biomedical Research, Institute of Primate Translational Medicine, Kunming University of Science and Technology, Kunming 650500, China; 4 Stem Cell Research Center, Peking University Third Hospital, Beijing 100191, China

**Keywords:** reproductive aging, hypothalamus, transcriptome, gender difference, housekeeping gene

## Abstract

Due to the current delay in childbearing, the importance of elucidating the underlying mechanisms for reproductive aging has increased. Human fertility is considered to be controlled by hormones secreted by the hypothalamic-pituitary-gonadal axis. To clarify the changes in hypothalamic gene expression with increasing age, we performed paired-end strand-specific total RNA sequencing for the hypothalamus tissues of rhesus. We found that hypothalamic gene expression in females was more susceptible to aging than that in males, and reproductive aging in females and males might have different regulatory mechanisms. Intriguingly, the expression of most of the hormones secreted by hypothalamus showed no significant difference among the macaques grouped by age and gender. Moreover, the age-related housekeeping genes in females were enriched in neurodegenerative disorders- and metabolic-related pathways. This study provides evidence that aging may influence hypothalamic gene expression through different mechanisms in females and males and may involve some nonhormonal pathways, which helps further elucidate the process of reproductive aging and improve clinical fertility assessment in mid-aged women.

## INTRODUCTION

Since 1950, the mean childbearing ages of both men and women have increased [1]. With age, both men and women show a natural decline in reproductive functions, including fertility and sex hormone status [2, 3]. Generally, women's fertility declines faster than men’s fertility, with a dramatic reproductive and endocrinological shift as well as the coming of menopause [4, 5]. In fact, reproductive aging and its sequelae are becoming a major health issue, as more women globally are delaying childbearing and life expectancy is increasing [6]. Therefore, studying reproductive aging is important and will help humans adapt to changes in childbearing age.

The hypothalamus is a brain region controlling reproduction, development, metabolism, homeostasis and circadian rhythm. A majority of physiological functions that decline with age, including reproduction, are extensively dominated and regulated by the hypothalamus. In mammals, the hypothalamus-pituitary-gonad (HPG) axis plays an essential role in the regulation of reproduction. Gonadotropin-releasing hormone (GnRH) neurons located in the hypothalamus release GnRH into the portal vessel in a pulsatile manner [7]. Secreted GnRH activates the pituitary to synthesize and release gonadotropins, such as follicle stimulating hormone (FSH) and luteinizing hormone (LH), to control gonad function. Therefore, the biological process driving pulsatile GnRH release has long been considered the major regulator of mammalian reproductive function [8, 9]. Moreover, some hormone-free pathways mediated by hypothalamus and involved in reproductive aging were observed in previous studies.

Rhesus macaque (*Macaca mulatta*) is one of the most common nonhuman primate animals used in neuroscience studies [10] and is phylogenetically close to humans [11]. Moreover, the brain structure and function of rhesus macaques are more similar to those of humans than other species, making it an ideal model to assess the mechanisms of the human brain [12]. Given the similarity between macaques and humans, analysis of the gene expression of the hypothalamus of the rhesus macaque could facilitate a better understanding of human reproductive aging. However, most of the current studies that have investigated the relationship between the hypothalamus and reproductive aging are limited by species and hypothalamus collection.

This is the first study to explore how the hypothalamus correlates with reproductive aging using rhesus macaque. In this study, we performed paired-end strand-specific total RNA sequencing for the hypothalamus tissues of young and middle-aged rhesus macaques to determine whether there are any changes in hypothalamic gene expression with increasing age.

## RESULTS

### Highly expressed genes in the hypothalamus of rhesus macaques

**Figure 1:**
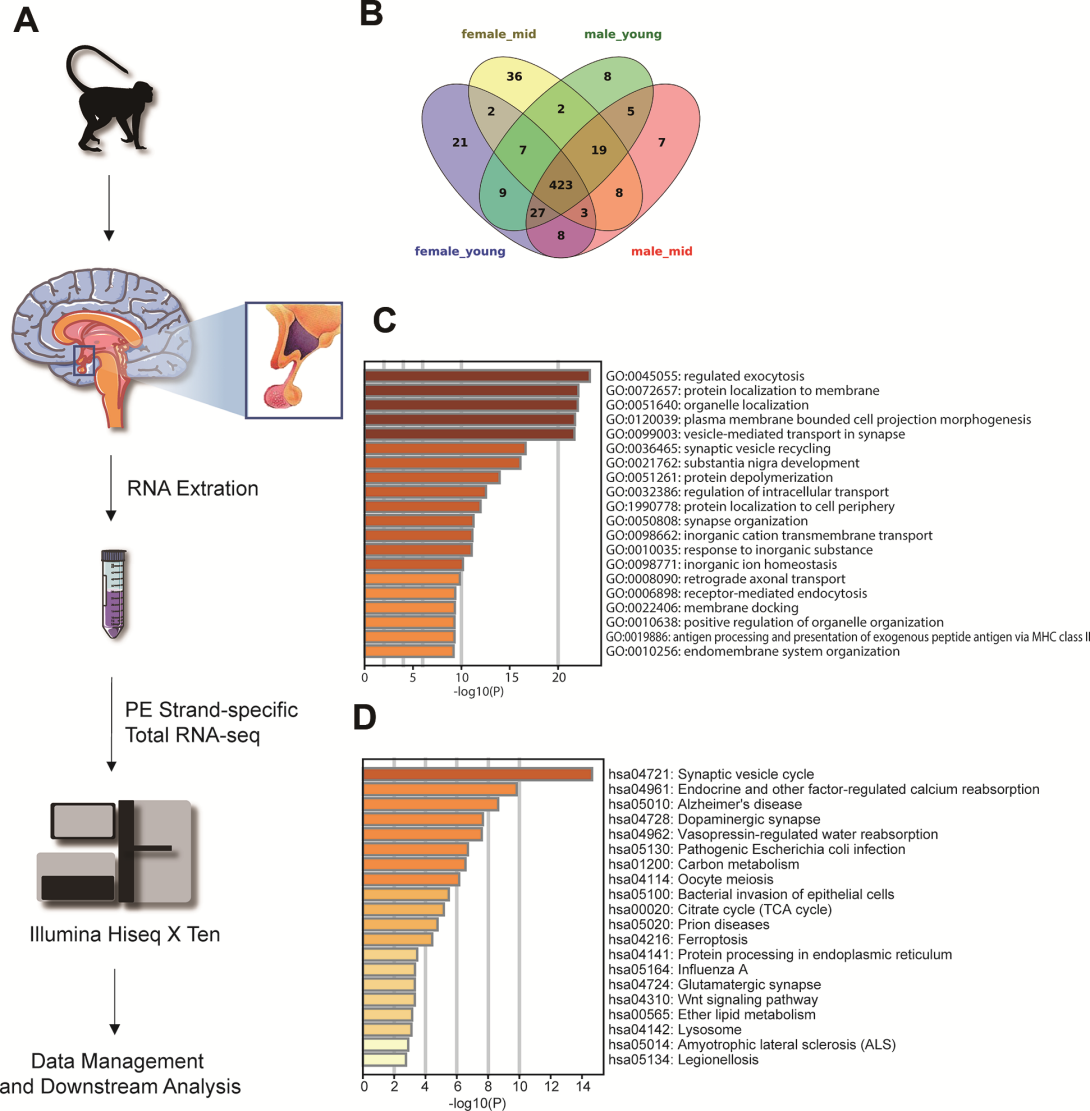
**Experimental design and highly expressed genes in the hypothalamus of rhesus macaque.** (**A**) Diagram of the experimental process. (**B**) The overlap of the top 500 genes in each group. (**C**, **D**) The GO biological process and KEGG pathway enrichment of the 423 overlapping genes in Figure 1B.

To elucidate the gene expression levels in the hypothalamus of rhesus macaques, we performed paired-end strand-specific total RNA sequencing (see Methods) for the hypothalamus tissues of young (not older than 5 years) and middle-aged (older than 5 years) rhesus macaques; each group had two males and two females (9 hypothalamus samples total, 3 samples in the male_mid group) (Figure 1A). For each sample, we sequenced an average of 36.7 million raw reads (ranging from 27.7 to 46.4 million reads), with mapping rates of approximately 88.9% (ranging from 85.9% to 90.3%, sequencing quality statistics in Supplementary Table 1). A total of 23651 genes were detected. Sequencing data are available at the GEO under accession GSE128537 (https://www.ncbi.nlm.nih.gov/geo/query/acc.cgi?acc=GSE128537).

To generate the gene expression profiles in each group, we compared the gene expression levels among the female_young, female_mid, male_young and male_mid groups (see Methods). Among the top 500 genes of each group, 423 were overlapped genes (Figure 1B–1D). Most of these overlapping genes were enriched in cellular functions such as regulated exocytosis, organelle localization, synaptic vesicle cycle, etc. Intriguingly, Kyoto Encyclopedia of Genes and Genomes (KEGG) pathway enrichment analysis of the overlapping genes showed enrichment in some neuropsychiatric diseases, such as Alzheimer’s disease (AD) and amyotrophic lateral sclerosis (ALS), in addition to oocyte meiosis. The Gene Ontology (GO) biological processes of the top 500 genes in each group are shown in Supplementary Figure 1. For instance, compared with other groups, the top genes in female_mid group showed enrichment in chemical synaptic transmission, which might indicate a more robust function of extracellular communication in the hypothalamus of female middle-aged macaques.

### Strong gender differences in hypothalamic gene expression during aging

**Figure 2:**
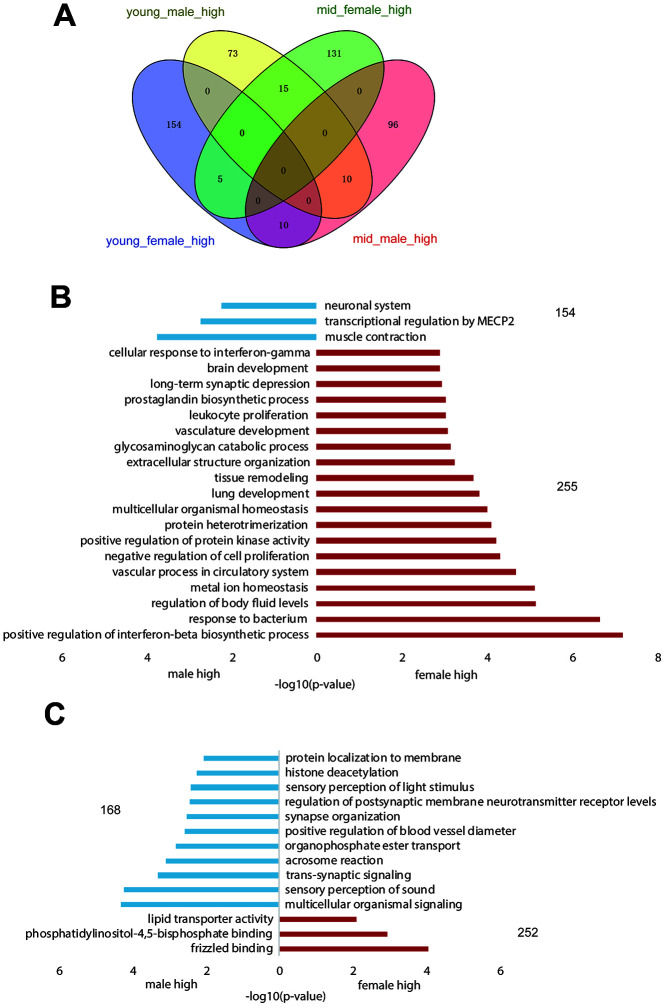
**Gender-related differentially expressed genes in the hypothalamus of rhesus macaque**. (**A**) The overlap of highly expressed genes in the rhesus macaque hypothalamus grouped by age and gender. (**B**, **C**) The GO functional enrichment of sex-related differentially expressed genes in the hypothalami of young (**B**) and middle-aged (**C**) macaques (red: upregulated genes in females; blue: upregulated genes in males).

We compared gene expression between the hypothalami of male versus female rhesus macaques and young versus middle-aged rhesus macaques (see Methods) to identify potentially sex-related genes (Figure 2A, Supplementary Datas 1–4) and age-related genes (Supplementary Datas 5–8). The GO functional enrichment results of differentially expressed genes by sex are shown in Figure 2B, 2C. The number of female_high genes was higher than that of male_high genes in both the young-aged and middle-aged samples (255 vs. 154 and 252 vs. 168, respectively), indicating that gene expression was more active in the hypothalami of female than male rhesus macaques.

**Figure 3:**
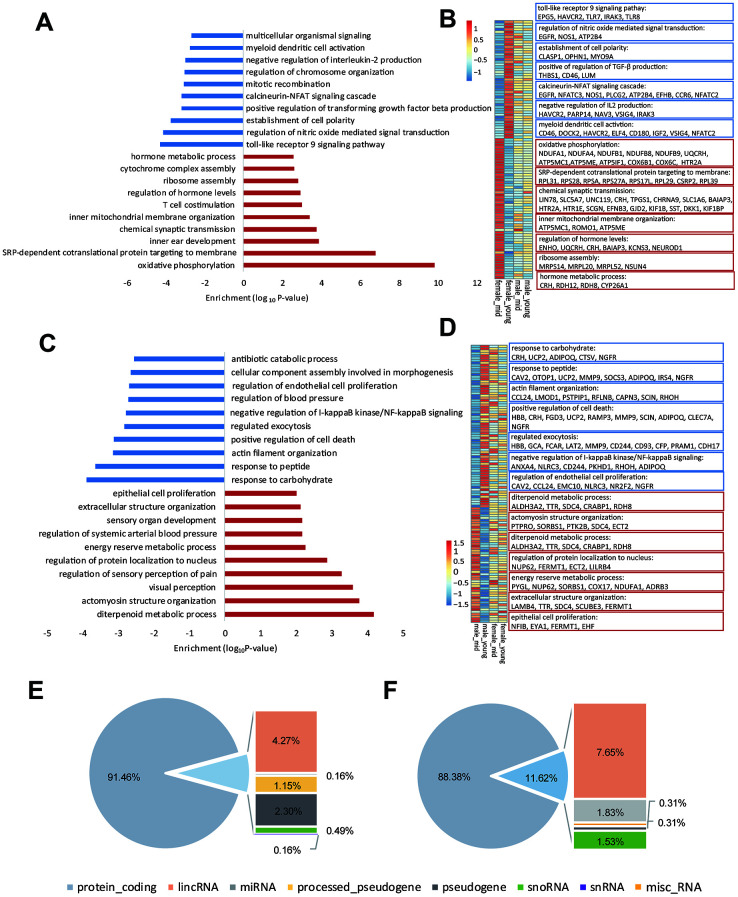
**Age-related differentially expressed genes in the hypothalamus of rhesus macaque.** (**A**–**C**) GO functional enrichment of age-related differentially expressed genes in the hypothalami of females (**A**) and males (**C**) (red: upregulated genes in middle age; blue: downregulated genes in middle age; each shows top 10 items). (**B**–**D**) Heatmaps of genes enriched in the items of (**A**, **C**). (**E**, **F**) Gene types of age-related differentially expressed genes in the hypothalami of females (**E**) and males (**F**).

**Figure 4:**
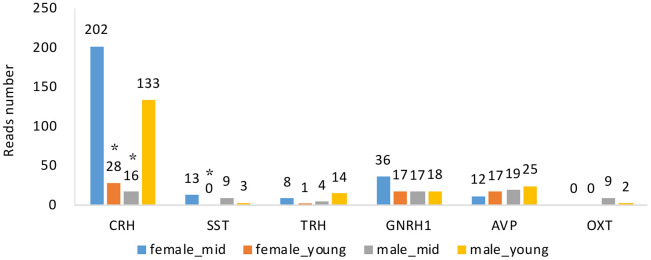
**Hormone gene expression of the hypothalamus of rhesus macaque in each group.** CRH, corticotropin releasing hormone; SST, somatostatin; TRH, thyrotropin-releasing hormone; GNRH1, gonadotropin-releasing hormone 1; AVP, arginine vasopressin; OXT, oxytocin. *, compared with female_mid group, p value < 0.05.

As shown in Supplementary Table 2, the difference in gender-related differentially expressed genes between the young and middle-aged samples was 11 (409 vs. 420, Supplementary Table 2), while the difference in age-related differentially expressed genes between female and male samples was 276 (605 vs. 329), which revealed strong gender differences in hypothalamic gene expression during aging. The number of age-related differentially expressed genes in females was 605, while the number in males was 329. As proved by previous studies, sex influences the rate of aging and the responses to many antiaging interventions in animal experiments. And we hypothesized that the gene expression in the hypothalamus of female macaques might be more susceptible to aging than that of males [13, 14]. GO functional enrichment of age-related differentially expressed genes in the female and male hypothalamus samples are shown in Figure 3A, 3C, and genes enriched in the items in Figure 3A, C are shown in Figure 3B, 3D, respectively. As shown in Figure 3B, 3D, corticotropin releasing hormone (CRH) was upregulated in the female hypothalamus during aging but downregulated in males, which was in accordance with the results of the hormone gene analysis below (Figure 4). These age-related differentially expressed genes of the female and male macaque hypothalamus samples were clustered into different functions, suggesting that the reproductive aging of female and male rhesus macaques might be regulated by different mechanisms and pathways.

To explore the reason for the strong gender differences in hypothalamic gene expression during aging, we analyzed the gene types of age-related differentially expressed genes in the female and male hypothalamic (Figure 3E, 3F). Importantly, in addition to the protein coding genes, lincRNAs accounted for a considerable proportion of these age-related differentially expressed genes, suggesting that lincRNAs might be involved in the process of reproductive aging. Moreover, we performed western blotting to 8 age-related differentially expressed genes in females (Supplementary Figure 2A, quantification of western blot in Supplementary Table 4), and the results of western blots were consistent with the differentially expressed gene lists (Supplementary Data 1–8) and the heatmaps shown in Figure 3B.

### Hormone gene expression and lincRNAs

Changes in hormone levels are essential in reproductive aging [15]. To determine the changes in hormone gene expression with aging, we compared the RNA levels of *CRH*, somatostatin (*SST*), thyrotropin-releasing hormone (*TRH*), *GNRH1*, arginine vasopressin (*AVP*) and oxytocin (*OXT*) among the 4 groups (see Methods). We analyzed the expression of *GNRH1* instead of *GNRH2* since *GNRH1* exerts its control in mammalian reproduction by stimulating the synthesis and release of LH and FSH [16]. By searching the differentially expressed gene lists (Supplementary Data 1–8), we found that apart from *CRH* and *SST* (p value < 0.05), the gene expression of hormones secreted by the hypothalamus did not show any differences among the groups (Figure 4). The expression level of *GNRH1* was slightly higher in the female_mid group than in the female_young group, but the difference was not statistically significant (p value > 0.05). We also performed qPCR to CRH and SST to confirm our the mRNA expressions of CRH in female_young and male_mid group were significantly lower than that in female_mid group. And mRNA expression of SST in female_young group was significantly lower than that in female_mid group. Thus, we inferred that CRH and SST were crucial in reproductive aging. The role of the HPA axis (containing CRH) in aging has been proven in some studies [17], but further research is needed to explore its roles in reproductive aging.

LncRNAs are considered to be essential in the development and function of the central nervous system (CNS) [18]. LincRNAs comprised the major set of lncRNAs. We identified lincRNAs in the hypothalamus with low coding potential and a length > 200 nt (see Methods). Next, we compared lincRNA expression among the rhesus macaques grouped by age and gender and revealed differences among the groups. Differentially expressed lincRNAs were found between female and male groups, and young- and middle-aged groups (as shown in Supplementary Figure 4), however, we could not elucidate the function and interaction of them since lncRNAs were poorly represented in the current annotation of the rhesus macaque genome. As the important role of lincRNAs in CNS development and function, we supposed that the effect of age on hypothalamic gene expression might be influenced by post-transcriptional regulation mediated by lincRNAs. Overall, the function of lincRNAs have been poorly elucidated in current studies of the rhesus macaque hypothalamus, and further investigation iswarranted.

### Housekeeping gene expression in the hypothalamus

Hormone gene expression levels, especially those of reproductive hormones, are believed to change gradually during the process of aging. According to the published literature, age diminishes hypothalamic GnRH secretion in males [15] but enhances GnRH secretion in females [9]. However, hormone expression analysis in this study revealed no differences in the hormone gene expressions (including GNRH1) among the groups, except for *CRH* and *SST*. This finding was incompatible with previous studies, since GnRH was considered as a major hormone correlated with reproductive function, and the decrease of GnRH neurons and the increase of GnRH secretion during aging were revealed by previous studies [9, 19]. We inferred that housekeeping gene expression might vary among the groups, which resulted in differences in protein translation capability and hormone secretion levels. Moreover, it was assumed that additional pathways involved in reproductive aging might be associated with hypothalamic housekeeping genes.

**Figure 5:**
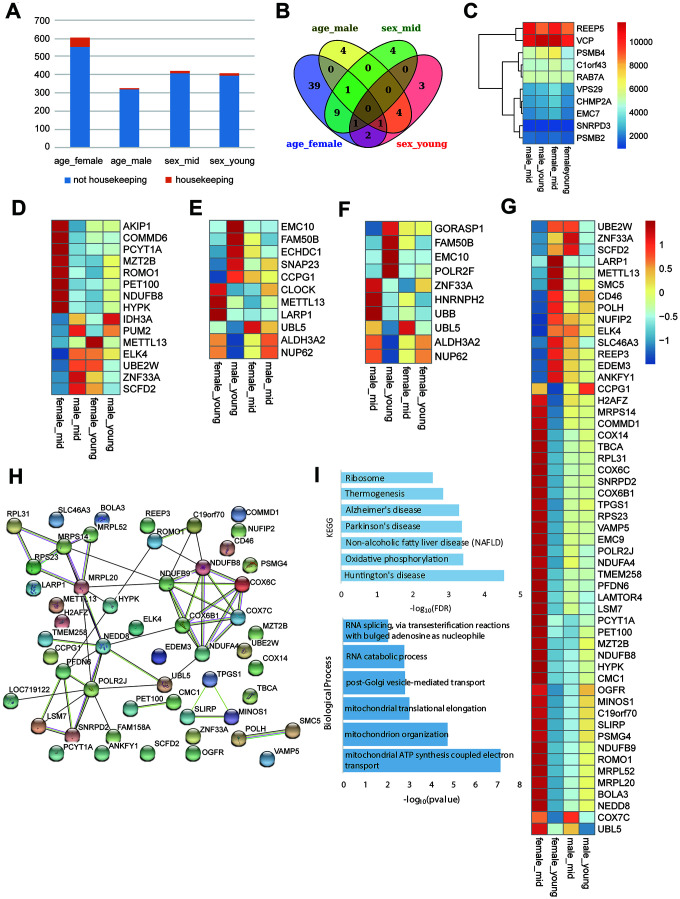
**Housekeeping gene expression in the hypothalamus.** (**A**) Proportion of housekeeping genes in differentially expressed genes (p value < 0.05). (**B**) The overlap of differentially expressed housekeeping genes according to age and gender. (**C**) The heatmap shows an example of stably expressed housekeeping genes. (**D**–**G**) The heatmap represents the differentially expressed housekeeping genes of middle-aged females vs. males (**D**), young-aged females vs. males (**E**), middle-aged vs. young-aged males (**F**), and middle-aged vs. young-aged females (**G**). (**H**) The protein interactions of differentially expressed housekeeping genes of middle-aged vs. young-aged females. (**I**) The KEGG pathway enrichment and GO biological process of differentially expressed housekeeping genes of middle-aged vs. young-aged females.

To investigate the role of housekeeping genes in reproductive aging, we compared the proportion of housekeeping genes in the differentially expressed genes among the rhesus macaques grouped by age and gender. As shown in Figure 5A, the proportion of housekeeping genes was higher in the age_female comparison than in the other comparisons, revealing that housekeeping gene expression in females was more susceptible to age than that in males. Venn diagram analysis showed that the number of differentially expressed genes in the age_female group was much higher than that in the other group (39 vs. 4/4/3). Further analysis of the differentially expressed housekeeping genes among the 4 groups revealed that the expression of the 68 housekeeping genes varied with gender and age (Figure 5A, 5B). We also performed qPCR and western blotting to the top 6 differentially expressed housekeeping genes between female_young and female_mid groups (Supplementary Figure 2B, 3B, quantification of western blot in Supplementary Table 4), and the expression changes of these genes were consistent with the heatmap shown in Figure 5G. Moreover, these age-related housekeeping genes in the female hypothalamus were highly enriched in neurodegenerative disorder- and metabolism-related pathways, such as AD, Parkinson’s disease, mitochondrial and ribosomal functions, as well as oxidative phosphorylation (Figure 5I). The protein interactions of differentially expressed housekeeping genes between the hypothalami of middle-aged and young female macaques were quite complicated. As shown in Figure 5H, NDUFB9, NDUFB8, NDUFA4, COX6B1, COX6C and COX7C compromised an interaction network with reaction, transcriptional regulation and posttranslational modification. These proteins participate in cellular respiration, and interact with some proteins mediating mitochondrial function, metabolism and ROS increasing (MRPL20, MRPS14, UBL5, ROMO1, etc.).

## DISCUSSION

In this study, we analyzed the transcriptome of the hypothalami in healthy rhesus macaques with RNA sequencing and summarized changes in gene expression during aging as well as the differences between males and females. We found strong gender differences in hypothalamic gene expression during aging as well as the potential influence of lincRNAs in this process. Moreover, this study revealed novel changes in hormone and housekeeping gene expression in the hypothalamus, which might indicate a critical role of nonhormonal regulation in reproductive aging.

Age had a greater effect on female hypothalamic gene expression than male hypothalamic gene expression since changes in gene expression in the male hypothalamus were much smaller than those in females during aging. Moreover, GO functional enrichment of age-related differentially expressed genes showed that reproductive aging of female and male rhesus macaques might have different regulatory mechanisms. This phenomenon might result from the fact that female fertility is the first to be adversely affected by aging in mammals, occurring decades prior to the deterioration of general physiological functions [6] and involving multiple pacemakers [20]. The results of this study are in accordance with the hypothesis that during the process of reproductive aging, females dominate and males cooperate. However, some researchers argued that reproductive aging is the outcome of male-female interactions [21], and the complexity of male reproductive senescence should also be considered.

Female reproductive aging is understood as a change in the hormone axis, mainly in the HPG axis. In women, the overall level of GnRH increases with aging [22]. Previous studies demonstrated that the decrease in estrogen levels, the depletion of ovarian follicles and the loss of negative feedback are responsible for this increase in GnRH secretion [9]. However, in this study, little difference was found in hormone gene expression between the hypothalami of young- and middle-aged female macaques. Importantly, housekeeping gene expression varied among groups, with a significant difference between young-aged females and middle-aged females. This finding suggested a regulation of the hypothalamic level in reproductive aging in addition to the HPG axis. In recent years, some studies have revealed nonhormonal pathways involved in female reproductive aging. Mitochondria are thought to play a key role in this process [23]. The compromised mitochondrial stress response was demonstrated to contribute to age-related accumulation of damaged proteins, reduced oxidative phosphorylation, and increased reactive oxygen species (ROS) production [24]. Gil Mor et al. also proposed a possible role for free radicals in hypothalamic reproductive aging [25]. Such a mechanism is supported by evidence that vitamin E, which can block estrogen-induced free radicals in the arcuate nucleus of the hypothalamus, delays the onset of constant estrus in aging female rats, and melatonin could protect oocytes from oxidative stress [26, 27]. Moreover, a connection between reproductive aging and AD is possible since reproductively senescent females were found to have higher amyloid-β (Aβ) formation and AD-like brain changes [28]. In this study, differentially expressed housekeeping genes were enriched in mitochondrial pathways, oxidative phosphorylation and some neurodegenerative disorders, such as AD, which supported the above mechanisms.

Other recent studies revealed the underlying basis of senescence, involving hypothalamic immunity mediated by IkB kinase-b (IKK-b), nuclear factor kB (NF-kB) and related microglia-neuron immune crosstalk [29]. Naturally, the "hypothalamic microinflammation" theory was promoted [30, 31]. Sirtuins were also demonstrated to be critical in governing multiple physiological functions in the brain, especially the hypothalamus [32]. Very recently, a non-neuronal mechanism regulating aging in the hypothalamus was further discovered, revealing that exosomal miRNAs released by hypothalamic neural stem/progenitor cells exert endocrine control over the speed of systemic aging [33, 34]. The findings above provide a new perspective on the role of the hypothalamus in aging and challenge the previous concept that the hypothalamus regulates reproductive function solely through the HPG axis.

There are some studies on aging brains of various species. Methodios et al. performed a single-cell transcriptomic analysis of aging mouse brains and highlighted age-related changes in cellular pathways and processes, which were also enriched in ribosome biogenesis [35]. A study by Kristofer et al. also revealed enrichment of genes in ribosomes, which slightly declined with age. During aging, genes involved in oxidative phosphorylation and mitochondria showed the most rapid declines [36]. Intriguingly, in this study, the KEGG analysis of differentially expressed housekeeping genes between the hypothalami of middle-aged and young-aged females was also enriched in ribosome pathways and oxidative phosphorylation. Xiao Xu et al. compared gene expression during aging in different cell types and found that the specific molecular consequences of aging were different between the cell types, and that genes downregulated with age were enriched for synaptic genes [37].

Overall, this study revealed strong gender differences in the gene expression levels of the hypothalami of rhesus macaques during aging. The hypothalamus of the female macaque was more susceptible to age than that of males, and different mechanisms and pathways might contribute to the changes in hypothalamic gene expression in males and females. In addition to the hormone genes expressed in the hypothalamus, more attention should be paid to housekeeping genes and the underlying mechanisms that might be involved in reproductive aging.

## MATERIALS AND METHODS

### Animals

In this study, 8 rhesus macaques with balanced age and sex were used. The 8 rhesus macaques were all from a single source, having similar rankings in the group and originating in South China. The macaques lived in groups outdoors and were all in good health. Individuals with the highest or lowest rankings, or in ovulation period were excluded. The rhesus macaques were grouped into young and middle-aged groups, and each group contained two males and two females. The mean ages of young-aged (not older than 5 years) females, middle-aged (older than 5 years) females, young-aged males and middle-aged male macaques were 4.1 y, 9.9 y, 3.9 y and 10.0 y, respectively (the specific age of each animal was shown in Supplementary Table 1). All experimental procedures complied with the Animal Ethics Procedures and Guidelines of the People's Republic of China. This study was approved by the Animal Ethics Committee of Peking University Third Hospital.

### Hypothalamus sample collection

The procedure of animal sacrifice and brain harvest was standardized. To minimize the ischemic time, we immediately harvested the brain of each individual when the rhesus macaque was euthanized. The brain was then cooled to 4°C. For each of the rhesus macaques, the brain was dissected into 52 regions. After the removal of frontal lobe, parietal lobe, occipital lobe, temporal lobe, limbic cortex and thalamus, the hypothalamus was carefully collected. The brain was dissected by one professional animal anatomist with thorough knowledge of the rhesus macaque brain structures and the whole process of hypothalamus dissection was completed in about 20 minutes. For each hypothalamus, 1 or 2 samples with size of approximately 2 to 3 mm^3^ were removed. For 7 of the 8 rhesus macaques (#4, #10, #7, #8, #6, #11, #2), 1 hypothalamus samples were collected and for the remaining one (#3), 2 hypothalamus samples were collected (as shown in Supplementary Table 1). The dissected samples were stored in liquid nitrogen before RNA extraction. The brain dissection and hypothalamus collection were completed with reference to the anatomical landmarks in the Brain Maps [38] (available at http://www.brainmaps.org) as well as the rhesus macaque brain atlas (A Combined MRI and Histology Atlas of the Rhesus Macaque Brain in Stereotaxic Coordinates 2^nd^ Edition).

### RNA-seq library preparation

For RNA sample preparations, a total amount of 3 μg RNA per sample was used as the initial material. First, RQ1 DNase (Promega) was applied to remove DNA after RNA extraction. Second, 1% agarose gels were used to detect RNA degradation and contamination. Then, the Kaiao K5500® Spectrophotometer (Kaiao, Beijing, China) was applied to assess the purity of the RNA samples. Subsequently, the RNA integrity and concentration were assessed using the RNA Nano 6000 Assay Kit of the Bioanalyzer 2100 system (Agilent Technologies, CA, USA). Finally, ribosomal RNA was removed using Epicentre Ribo-ZeroTM Gold Kits (Human/Mouse/Rat) (Epicentre, USA).

The RNA-seq library was generated following the manufacturer’s recommendations with varied index labels using the NEBNext® UltraTM Directional RNA Library Prep Kit for Illumina (NEB, Ipswich, USA). First, RNA fragmentation was conducted using NEBNext First Strand Synthesis Reaction Buffer at 94°C for 15 minutes. Second, first strand cDNA synthesis was performed with RNA fragments as a template and random hexamer primers. The second strand cDNA was then synthesized with buffer, dNTPs, DNA polymerase I and RNase H. The library fragments were purified with QiaQuick PCR kits and eluted with EB buffer. Terminal repair and sequencing adaptor addition were then carried out. For selection of cDNA fragments preferentially 300 bp in length, the library fragments were purified with agarose gel electrophoresis, and the second strand of cDNA was digested by UNG enzyme. Finally, PCR was performed, and the desired products were retrieved by agarose gel electrophoresis.

### RNA-seq

The library was sequenced on the Illumina HiSeq X Ten (HiSeq x Ten Reagent Kit v2.5) platform. Then, FastQC (v0.11.4) was utilized to conduct read-level quality control of the FASTQ files generated by the Illumina workflow before mapping. For samples with poor quality, quality trimming was performed by Trim Galore (v0.4.4, Quality Phred score cut-off: 30). Subsequently, HISAT2 [39] (v2.0.5, default parameters) was applied to map the reads with the Mmul 8.0.1 reference genome and the Mmul 8.0.1.91 transcriptomic annotation GTF (Ensembl, available at http://ftp.ensemblorg.ebi.ac.uk/pub/release-91/gtf/macaca_mulatta/Macaca_mulatta.Mmul_8.0.1.91.gtf.gz). Read counts of the genes were generated by HTSeq (v0.6.1), and the read count matrix for transcripts was assembled by prepDE.py script from the StringTie (v1.3.4, with parameter -e) package. For evaluation of RNA integrity at the transcript level, TIN (transcript integrity number) was calculated for each sample using tin.py from RSeQC (v2.6.4) tools. The median TIN score (medTIN) of all transcripts was used for measurement [40].

A total of 23651 genes were detected and used in downstream analysis. Normalization of the read count matrix and identification of differentially expressed genes were achieved by DESeq2 (v1.18.1). Genes with FDR < 0.05 and fold-change > 2 compared to other groups (pairwise comparison) were used for functional annotation.

### Analysis of age- and sex-related differentially expressed genes

To generate the profile of gene expression in the hypothalamus, we selected the top 500 genes of each group (female_young, female_mid, male_young, male_mid) and found 423 overlapping genes. GO enrichment of biological process and KEGG enrichment of these overlapping genes and genes in each group were conducted in the Database for Annotation, Visualization and Integrated Discovery (DAVID) v6.8 website (available at https://david.ncifcrf.gov/). (performed by CapitalBio Technology, Beijing)

Next, we selected young_male_high genes, young_female_high genes, mid_female_high genes and mid_male_high genes with DESeq2 (v1.18.1). For instance, genes with higher expression in female_young than male_young were considered young_male_high genes, and genes with higher expression in female_mid than female_young were considered mid_female_high genes. GO functional enrichment analysis of these genes was conducted using the DAVID v6.8 website. Heatmaps of these genes were drawn through R Studio (v3.5.1).

### Analysis of lincRNAs

Novel transcripts located in the intergenic region or antisense to known genes were selected. The minimal distance between candidate lincRNAs and the nearest protein-coding genes (annotated by Mmul 8.0.1.91) was set to 1 kb. CPAT (Coding Potential Assessment Tool) or CPC2 (Coding Potential Calculator) was utilized to assign the candidate lincRNAs as noncoding. Additionally, transcripts shorter than 200 bases and consisting of one single exon were excluded from the final list of lincRNAs.

We analyzed differentially expressed lincRNAs among the rhesus macaques grouped by age and gender. For age-related lincRNAs, for example, we recognized age_female_up genes with DESeq2 (v1.18.1) by comparing the lincRNA expression levels between young_female and mid_female groups, and genes with higher expression in the mid_female group than in the young_female group (p value < 0.05) were considered age_female_up genes. The identification of age_female_down genes, age_male_up genes and age_male_down genes was performed in a similar manner. For the analysis of gender-related genes, for instance, genes expressed at higher levels in the young_female group than in the young_male group were considered young_female_high genes. Next, the GO functional enrichment of these genes was conducted using the DAVID v6.8 website.

### Analysis of housekeeping genes

We identified 68 housekeeping genes of the hypothalamus and compared the expression levels among the 4 groups through DESeq2 (v1.18.1). For instance, differentially expressed housekeeping genes between female_young and female_mid were considered age_female genes, and differentially expressed housekeeping genes between female_mid and male_mid were considered sex_mid genes. Heatmaps that represented the differentially expressed housekeeping genes of middle-aged females versus males, young-aged females versus males, middle-aged versus young-aged males, and middle-aged versus young-aged females were generated by R Studio (v3.5.1). The KEGG enrichment and GO biological process enrichment of these differentially expressed housekeeping genes were conducted using the DAVID v6.8 website. Moreover, the protein interaction diagram of the differentially expressed housekeeping genes of middle-aged versus young-aged females was generated through the STRING v11 website [41] (available at http://string-db.org).

### Quantitative real-time PCR (qPCR)

qPCR was performed to analyse the mRNA expressions of the top 6 differentially expressed housekeeping genes between female_young and female_mid (COX6C, HYPK, CMC1, LARP1, METTL13, SMC5), and the differentially expressed hormone genes (CRH, SST). Specific primers were designed for the amplification of the target and housekeeping transcripts (Supplementary Table 3). The reverse transcription was performed using a cDNA Synthesis Kit (Thermo Scientific, Massachusetts, USA). The complementary DNA was then amplified in triplicate using the PowerUp SYBR Green Master Mix (Thermo Scientific), according to the manufacturer’s protocol. qPCR was then performed and cycle number beyond 35 was excluded. The levels of mRNA for each gene were calculated using the 2^-ΔΔCT^ method relative to the corresponding mRNA levels of female_mid group. The differences among the groups were calculated by one-way ANOVA analysis, and p value < 0.05 was considered as significant.

### Western blot

Briefly, protein quantification was conducted using a BCA Kit. Protein lysates were subjected to SDS-PAGE and subsequently electrotransferred to a polyvinylidene fluoride membrane (Millipore). The membrane was incubated with the indicated primary antibodies overnight at 4 °C and HRP-conjugated secondary antibodies for 1 hat room temperate, followed by visualization using the ChemiDoc XRS system (Bio-Rad). Quantification was performed with Gel Image ststem ver.4.00 (Tanon, china).

## Supplementary Material

Supplementary Figures

Supplementary Tables

Supplementary Data 1-8
