# Supplementary material for: Transcriptomic analysis reveals gender differences in gene expression profiling of the hypothalamus of rhesus macaque with aging
Source: Aging (Albany NY). 2020 Sep 27;12(18):18251–73. doi: 10.18632/aging.103682 (PMC7585077; doi:10.18632/aging.103682)
Supplement: Supplementary Figures [file aging-12-103682-s003..pdf]

SUPPLEMENTARY FIGURES

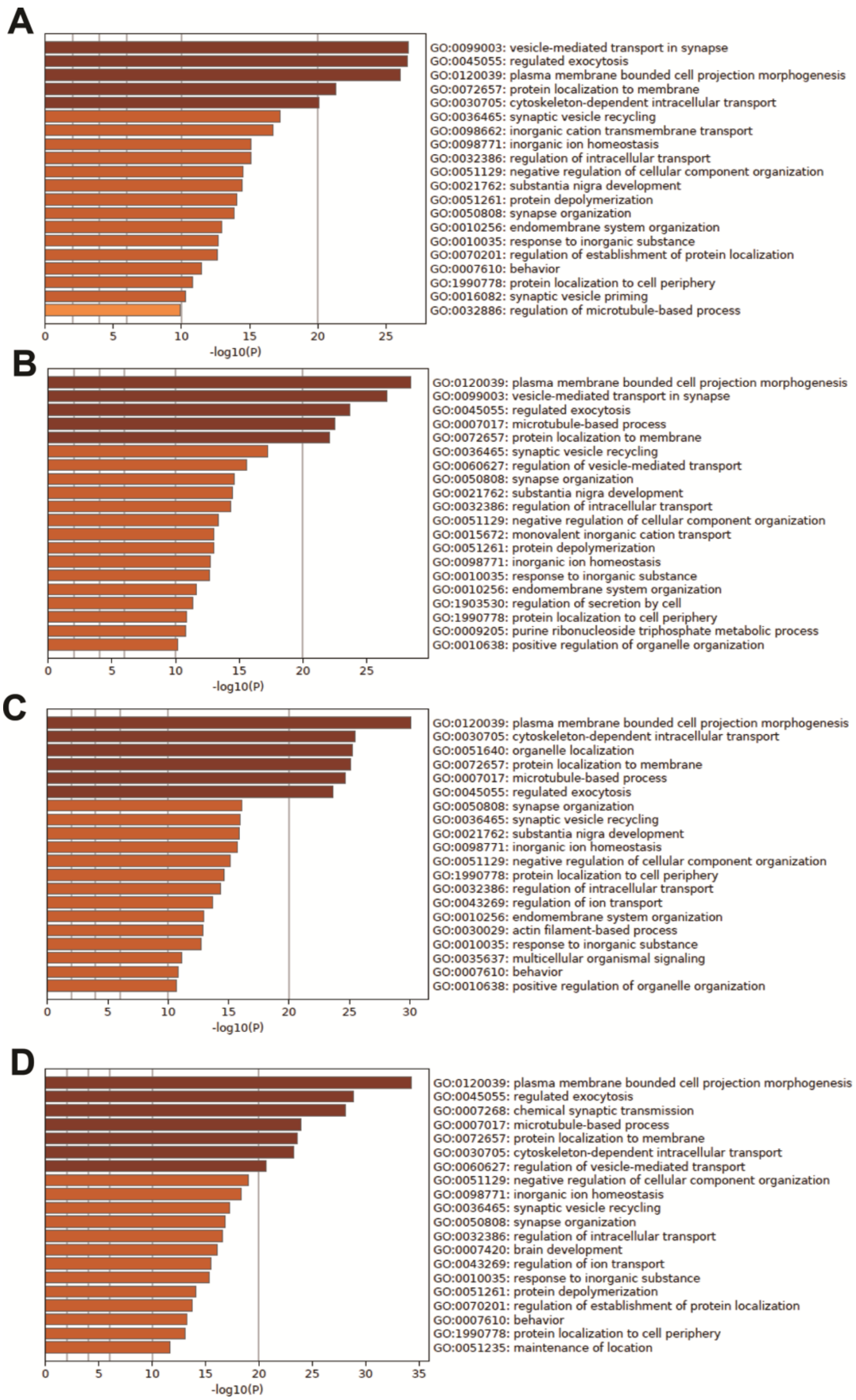

Supplementary Figure 1. The GO biological process of the top 500 genes in the hypothalamus of rhesus macaque. (**A**) The top 500 genes in the hypothalami of young-aged male macaques; (**B**) The top 500 genes in the hypothalami of middle-aged male macaques; (**C**) The top 500 genes in the hypothalami of young-aged female macaques; (**D**) The top 500 genes in the hypothalami of middle-aged female macaques.

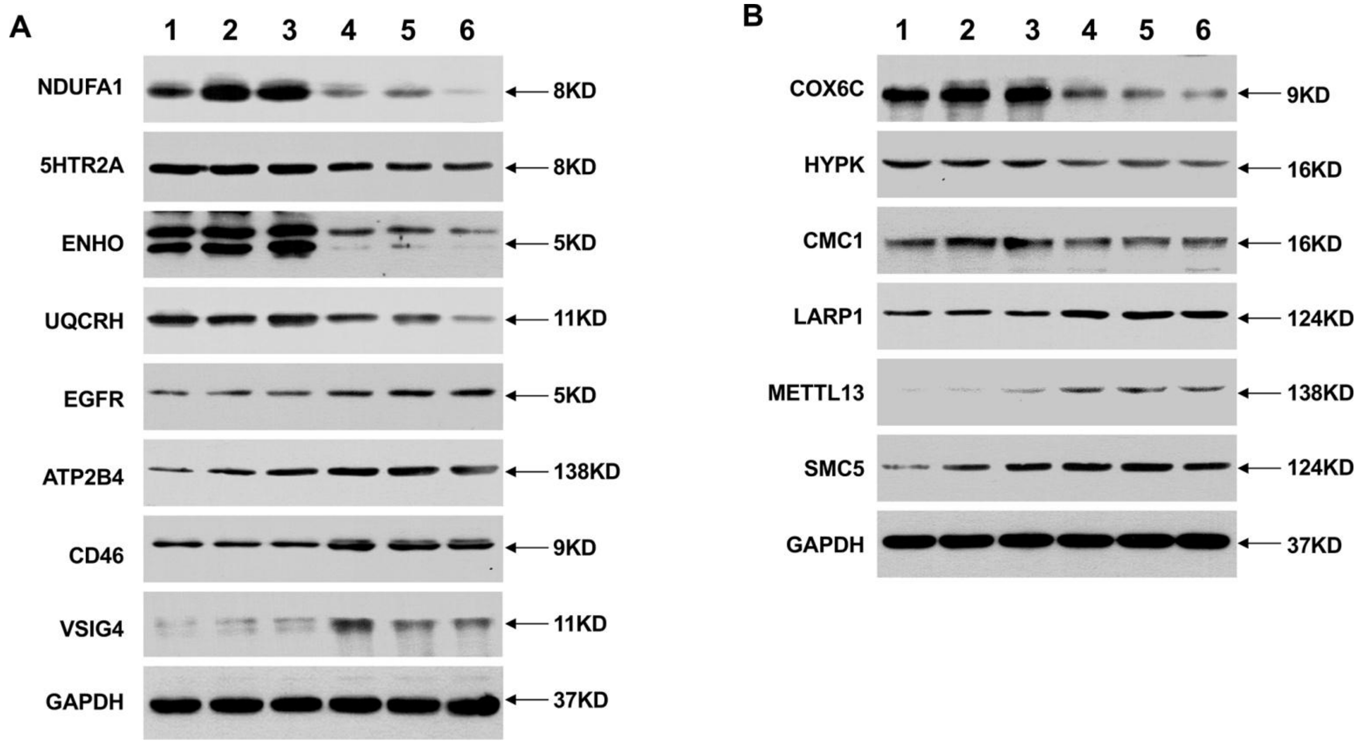

Supplementary Figure 2. Western blots of differentially expressed genes. (**A**) Age-related differentially expressed genes in female macaques. (Sample 1-3: female_mid; sample 4–6: female_young). (**B**) Age-related differentially expressed housekeeping genes in female macaques. (Sample 1-3: female_mid; sample 4–6: female_young).

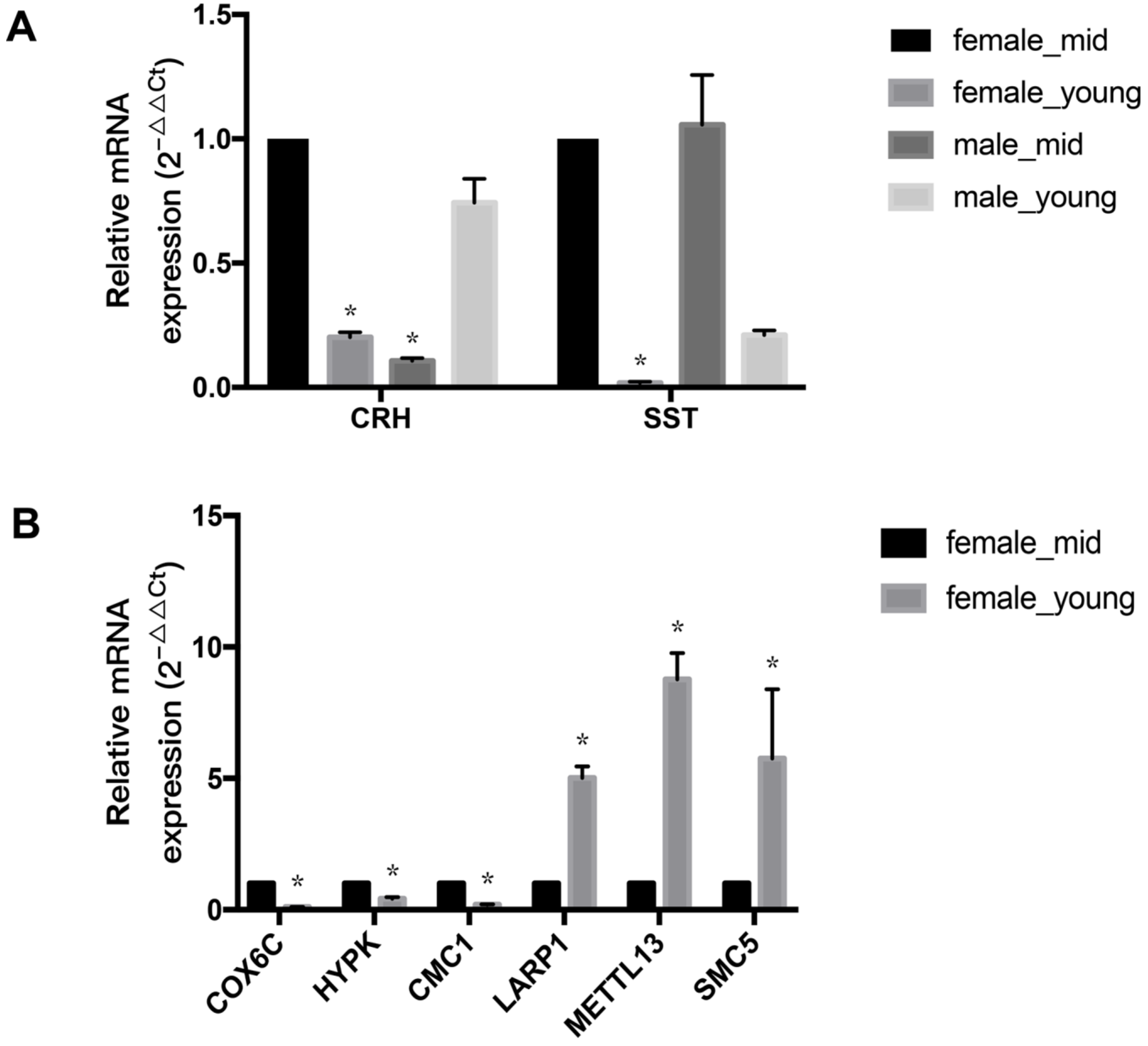

Supplementary Figure 3. Relative mRNA expression of hormone genes and differentially expressed housekeeping genes. (A) CRH, corticotropin releasing hormone; SST, somatostatin. *, compared with female_mid group, p value < 0.05. (**B**) *, compared with female_mid group, p value < 0.05.

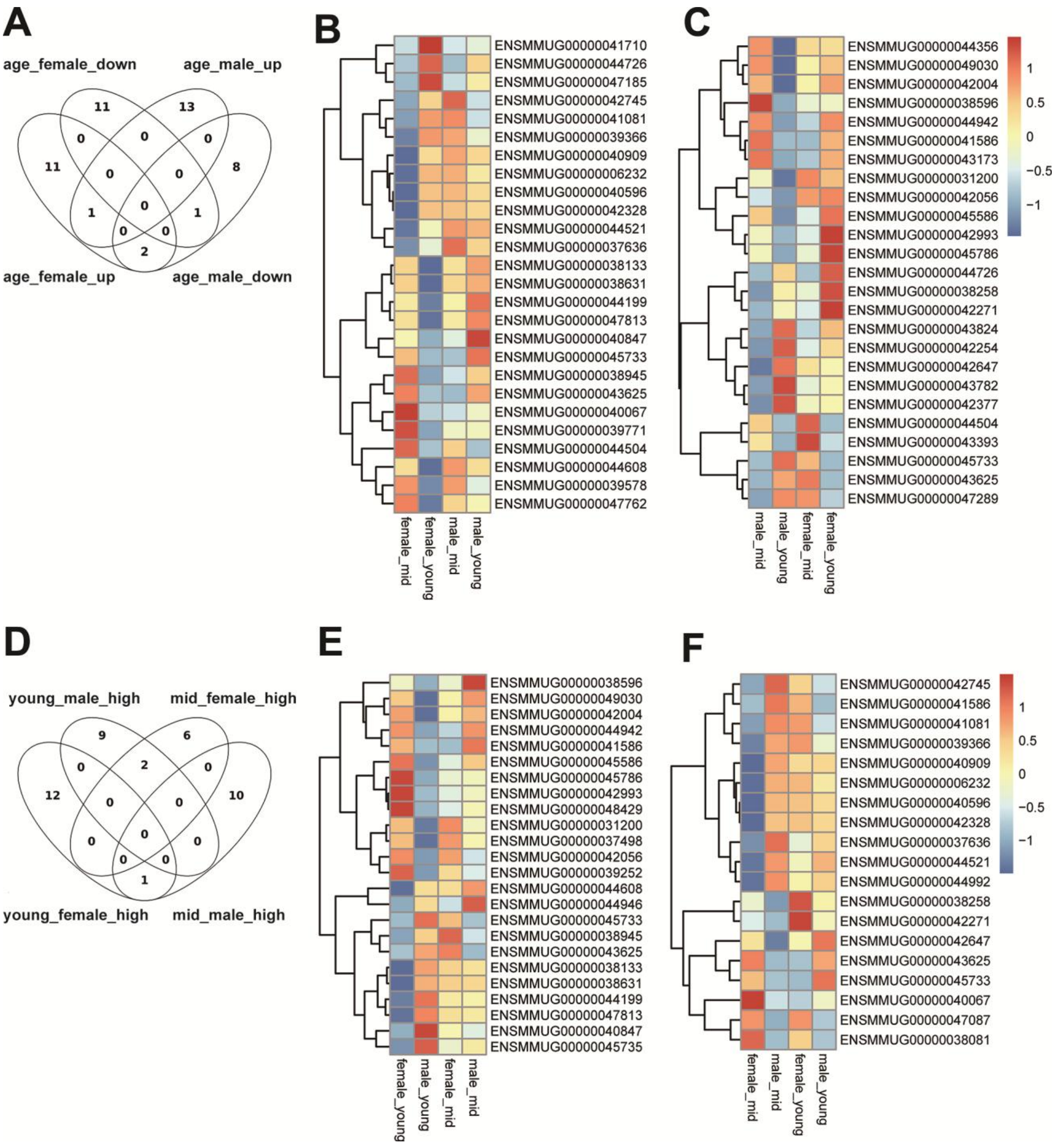

Supplementary Figure 4. The Venn diagrams and heatmaps of differentially expressed lincRNAs by age and gender. (**A**–**D**) The number of differentially expressed lincRNAs in the hypothalami of rhesus macaques grouped by age and gender. (**B**, **C**) (**E**, **F**) Heatmaps show the differentially expressed lincRNAs in the hypothalami of rhesus macaques grouped by age and gender.
