# Supplementary material for: Transcriptomic analysis reveals gender differences in gene expression profiling of the hypothalamus of rhesus macaque with aging
Source: Aging (Albany NY). 2020 Sep 27;12(18):18251–73. doi: 10.18632/aging.103682 (PMC7585077; doi:10.18632/aging.103682)
Supplement: Supplementary Tables [file aging-12-103682-s002..pdf]

SUPPLEMENTARY TABLES

Supplementary Table 1. The RNA sequencing quality of the hypothalamus of rhesus macaque.

Sample number	Raw reads	Trimmed paired	Aligned concordantly exactly 1 time (--dta)	Overall alignment rate	Age	Gender
#4	39,345,822	36,553,768	27,008,702 (73.89%)	85.92%	Young (3.2 y)	Female
#10	34,739,393	32,699,812	26,374,273 (80.66%)	90.08%	Young (5 y)	
#7	32,586,392	30,925,534	25,020,200 (80.90%)	90.26%	Young (4 y)	Male
#8	33,283,310	30,876,062	24,721,694 (80.07%)	89.66%	Young (3.8 y)	
#6	46,426,721	43,647,729	34,112,556 (78.15%)	88.34%	Middle (10 y)	Female
#11	43,444,130	41,253,888	32,722,384 (79.32%)	89.46%	Middle (9.8 y)	
#2	32,777,557	31,597,336	24,785,971 (78.44%)	88.30%	Middle (10 y)	Male
#3-1020	27,741,388	25,913,402	20,369,952 (78.61%)	88.90%	Middle (10 y)	
#3-1026	40,113,034	37,304,146	29,307,116 (78.56%)	88.88%		

Supplementary Table 2. The number of differentially expressed genes by sex and age (p value < 0.05).

	Female	Male	Number
Young	#4, #10	#7, #8	409
Middle	#6, #11	#2, #3	420
Number	605	329	

Supplementary Table 3. The primers used in RT-qPCR amplification.

Gene		Primer	Length
CRH	F	agtaccctcagcccttggat	131bp
	R	gctcttgttgaggttcccca	
SST	F	ccccagactccgtcagtttc	100bp
	R	gttgggttcagagagcagct	
COX6C	F	ggattcgtgctatccctggg	132bp
	R	accagccttcctcatctcct	
HYPK	F	gcagaaagccaaacaggagc	132bp
	R	cccatgtgttcccgtaagct	
CMC1	F	gcagaccagcatctcagaca	83bp
	R	tcagaacacctctctttggcc	
LARP1	F	ccaaccactgtcccagagtc	96bp
	R	ccgtgatgtctggcttgagt	
METTL13	F	ttttcatcatcccccagggc	110bp
	R	aagggccactgtaatcagcc	
SMC5	F	gccagtttctccctcaggac	104bp
	R	tctgtgcatttctgggggac	
GAPDH	F	tcgtcatcaatggaagcccc	136bp
	R	aaatgagccccagccttctc	

Supplementary Table 4. Quantification of western blots (Sample 1–3: female_mid; sample 4–6: female_young).

Gene	Sample 1	Sample 2	Sample 3	Sample 4	Sample 5	Sample 6
NDUFA1/GAPDH	0.82	1.43	1.29	0.22	0.34	0.16
UQCRH/GAPDH	0.91	0.98	1.02	0.61	0.53	0.25
COX6C/GAPDH	1.31	1.70	1.60	0.53	0.40	0.26
ENHO/GAPDH	1.28	1.50	1.44	0.51	0.54	0.30
ATP2B4/GAPDH	0.20	0.43	0.57	0.87	0.89	0.74
LARP1/GAPDH	0.39	0.39	0.37	0.73	0.78	0.64
HYPK/GAPDH	0.62	0.55	0.55	0.33	0.49	0.36
HTR2A/GAPDH	0.95	0.77	1.02	0.64	0.47	0.53
VSIG4/GAPDH	0.16	0.29	0.29	0.71	0.52	0.54
CD46/GAPDH	0.60	0.56	0.48	0.94	0.82	0.82
EGFR/GAPDH	0.34	0.29	0.28	0.59	0.67	0.61
METTL13/GAPDH	0.15	0.16	0.29	0.50	0.41	0.46
SMC5/GAPDH	0.12	0.32	0.48	0.61	0.65	0.62
CMC1/GAPDH	0.85	0.86	0.95	0.70	0.61	0.64
