# Supplementary material for: Transcriptomic analysis reveals gender differences in gene expression profiling of the hypothalamus of rhesus macaque with aging
Source: Aging (Albany NY). 2020 Sep 27;12(18):18251–73. doi: 10.18632/aging.103682 (PMC7585077; doi:10.18632/aging.103682)
Supplement: Supplementary Data 1-8 [file aging-12-103682-s001..docx]

**Supplementary Data**

**Supplementary Data 1. The differentially expressed genes between female_mid and male_mid macaques (female_mid higher than male_mid, p value＜0.05).**

NXN

RF02266

RF01881

OPRPN

AIF1

CRH

KCNS3

RPS4X

LGALS1

MYO1G

CTHRC1

POPDC3

NLRC3

ZNF541

BAIAP3

ATP5MD

PVALB

FBP2

LAMC3

UNC119

AKIP1

DPYSL2

PCP2

APOC1

VMO1

DPEP3

IZUMO2

CCL24

ARHGAP27

MZT2B

FGD3

FAM167B

COMMD6

NDUFB8

SLC1A5

SH2D2A

PBX4

RF00017

GZMM

OTOP1

TSTD1

CD48

UNC93B1

ZNF256

CCDC188

ERCC6L

HYPK

PDGFRL

TNNI1

OR2B11

FREM3

SPATA22

ABI3

PCYT1A

TMCO4

TMA7

PTER

HSD17B13

RAP1GAP2

ROMO1

SCGB3A1

PROSER2

ACOT6

BTNL3

TSPAN5

LY6H

TTPA

CD244

AGMO

NEIL3

NOD1

RF01880

CYP2A23

.

SULT1B1

ACCS

GPR6

MEIG1

GPR143

RSPO3

ST6GALNAC1

SCIN

WNT1

TTC9B

VWF

NGFR

PEX10

NUSAP1

PLEKHG4

FABP1

CFP

ATP5ME

SLC16A3

RHEBL1

EXOC1L

TMEM30B

TRIM7

INAFM1

PET100

SOX11

KCNJ1

PTGER1

ADAD2

PPP1R1C

BHLHE22

KYNU

CHIT1

RF00017

ITIH1

GLI1

MLPH

EFCC1

PRRT4

PRKCG

KCNG2

DIP2B

KCTD8

CD2

SLC22A6

COX7AH

CA8

SOCS3

DIRAS3

AIRE

HTR6

GYG2

ZAP70

RBP4

ZBTB42

DNPH1

LYPD6B

NRROS

TLR5

FNDC11

CCL8

BSPRY

SYNPR

AEBP1

RHOH

PHLDA2

CORO2A

KIF1B

SLC47A1

CHEK2

STAC3

TGM3

GPR101

C4orf48

NKAIN3

NRAP

C1orf53

RSPO1

PILRA

KLK7

**Supplementary Data 2. The differentially expressed genes between female_mid and male_mid macaques (female_mid lower than male_mid, p value＜0.05).**

DDX3Y

USP9Y

RPS4Y1

RPS4Y2

.

ZFY

EIF1AY

UTY

KDM5D

NLGN4Y

SYT10

ZNF33A

ANK2

ZIC4

PYGL

TBL1Y

ETNK1

UBE2W

PGAP2

DPP8

COL11A2

ERBIN

SCFD2

AQP6

TCF19

COLQ

NEB

IL25

NAALAD2

IDH3A

EHF

CRYBB2

MRPL39

USH2A

HMCN1

DAPP1

NOS1

RF01241

CHL1

ASXL3

CD5L

ESRRG

PLB1

FCRL6

KCNJ5

TCEANC

TENM1

CYB561D1

USP37

MTTP

RDM1

SOX5

TNNI3K

DNAAF4

EGFR

METTL13

UNC5C

EPG5

NFATC3

TRIM5

MTNR1A

MRC1

COL21A1

CACNB4

KIAA1147

MYO9A

SFN

STPG2

ARMH4

SLC14A1

FRMPD4

TRIM33

ZDHHC21

FCRL5

RALGPS2

CCBE1

ELK4

ROPN1B

PDYN

ATP8B4

ABCC11

FAM117B

RF02153

MESP2

SLC5A3

PCDH15

PLCD4

FRRS1L

BDKRB2

PLA1A

FAM198B

CNTN5

ADAM22

OTOA

ENPP4

DUSP27

RF00017

IGHM

RCOR1

ZSCAN20

PRR25

ZNF560

PRC1

SORCS1

IFT52

SCN2B

GABRB2

G3BP2

MME

PUM2

FAM102B

FREM2

SMR3B

REXO5

RF00017

RAB3C

RF00105

CDKL5

GABRA4

**Supplementary Data 3. The differentially expressed genes between female_young and male_young macaques (female_young higher than male_young, p value＜0.05).**

NFIB

RF02266

RF01881

LAMB4

ALDH3A2

TMEM117

CSF1R

SLC24A3

ABCC4

DPYSL2

EYA1

PREX2

PPIL6

HPGDS

ANGPTL1

HMCN1

COL1A2

PNMA6A

SLC6A11

SLC39A12

BBOX1

FSIP2

RBPJ

METTL13

KDM6A

CD4

GJB6

SORBS1

CYSLTR1

UBL5

FMR1

RF01880

NUP62

RF00322

MRC1

CD5L

NT5E

FCRL5

TLR4

ATP13A4

C6H5orf49

SDC4

IGF2BP1

LAMC3

MYBPC1

MMS22L

OLFML3

UPK3B

ERN2

NOV

AQP4

GZMB

HSD11B2

IGSF1

C5AR1

CNGA3

OAS2

CCDC129

ATP1B2

LCP2

GMNC

GLT8D2

TLR8

COX17

RF00397

TRIQK

POPDC2

AKAP3

MPEG1

SLC13A4

FNDC1

EDNRB

PLAC8

TLR7

CCBE1

SLC7A13

ARSJ

BMPR1B

ZFX

LOR

PXDC1

ST8SIA5

GJA1

MFAP5

TRPM3

FBLN1

SCUBE3

GPC3

PTPN14

TMEM72

NR0B1

C1QTNF6

C5

SCARA5

VSIG4

CRYGN

SLFN11

SLC7A10

FAM213B

ST8SIA4

PDC

KDELR3

MAGI1

ADRB3

APOBEC3G

EGFR

ESPNL

CLDN10

TLR3

LILRB4

CLOCK

GPR182

PIK3R5

RF00009

ELF4

MLLT3

HNF4G

KCNB2

F13A1

FCRL6

AVPR1B

SLC13A5

C21orf62

CIITA

ZNF556

RF02153

CELSR1

CD300LD

LUM

SSC5D

ARMC4

CXCR2

ITGA11

CCR6

SCGB3A2

FIBIN

DNAH6

PRKG1

PCDH12

NEUROD4

CD38

PNPLA7

ADAMTSL3

REL

SLC26A2

TBX4

TMEM245

ATP8B1

AXL

FZD10

PLEK2

RF00418

DUOXA1

PRELP

PTK2B

.

CDR1

NXF3

CUBN

STYK1

NFAM1

SELP

DCST1

C1orf115

PCSK9

PLA1A

LARP1

MANSC4

SSPO

PTGS1

**Supplementary Data 4. The differentially expressed genes between female_young and male_young macaques (female_young lower than male_young, p value＜0.05)**

DDX3Y

USP9Y

RPS4Y1

RPS4Y2

ZFY

.

EIF1AY

UTY

KDM5D

MYADML2

SPATA22

TBL1Y

FAM219A

SLC5A7

DUSP19

AP3M2

FAM50B

NLGN4Y

RF00181

DPEP3

TSPAN5

GTSF1

IFNL1

PVALB

SNAP23

.

SELENOW

PCSK1

ENC1

PCP2

CCPG1

RCAN3

AXDND1

F2

.

.

CTHRC1

CRH

CBLN3

OTOP1

TENM4

PDZK1

EMC10

FAM160A1

PDE1C

TGM5

GPR101

SAMD13

SKA1

CHRNA9

SPIN4

EIF4E1B

CAPN3

ZAP70

CD244

KCNK5

KCNA3

ECHDC1

MYL2

NR2E3

LECT2

SLC26A5

ACOT6

KCNJ1

MPZ

NEUROD1

FXYD6

ATP2A1

.

PERP

OPN3

TTF2

CALHM6

OR2B11

RF00599

MYH3

MET

ADIPOQ

CENPK

TTLL9

PIMREG

TNNI1

DTHD1

FRMPD1

CAVIN2

ITGB7

NRK

EFCAB11

TMEM202

RF00017

KRT8

.

SSTR4

GGN

PBX3

IL1A

RAB38

TMEM200A

EXO1

CNN1

GFRA4

CMTM8

OTOF

IL1RAPL2

**Supplementary Data 5. The differentially expressed genes between female_mid and female_young macaques (female_mid higher than female_young, p value＜0.05)**

MYADML2

NDUFA1

DUSP19

CD52

PCP2

SMDT1

MRPS14

PVALB

LGALS1

RPL31

MINOS1

NTHL1

LIN7B

SLC5A7

TMEM202

AP3M2

COX14

NDUFB9

ACOT6

TMEM258

BRI3

ENHO

SPATA22

RPS29

SNRPD2

H2AFZ

COL6A1

BOLA3

ATP5MPL

NDUFB8

LAMTOR4

CTHRC1

RPS28

UQCRH

KCNJ1

SVBP

UNC119

NEDD8

IFNL1

ATP5MC1

ROMO1

LY6H

FAU

.

POLR2J

TMA7

AIG1

SELENOH

C6orf125

RPL39

ARHGEF39

COMMD1

MRPL20

GSTM3

GAL

TXN

LSM7

DPEP3

CRH

MIR6506

NDUFA4

AXDND1

RPSA

RPS27A

MZT2B

RFLNA

VAMP5

RPS17L

RDH12

KRTCAP2

FUOM

TUBA4B

CDKN2D

ASGR1

ELOB

TBCA

HYPK

ATP5MD

RPS23

MRPL52

LARP6

NME7

CYP3A5

COX6B1

ATP5ME

.

C19orf70

TPGS1

FAM219A

RF00181

COX6C

SLIRP

SLC1A5

YJEFN3

ANKRD34B

TSTD1

CHRNA9

SLC1A6

BAIAP3

PFDN6

OCIAD2

ATP5IF1

PSMG4

NDUFAF8

TTC9B

SNX16

KCNS3

OGFR

.

RDH8

.

IFI27L2

HEBP2

PIMREG

EMC9

CD244

OPRPN

C11H12orf57

.

SLC26A5

FABP1

COX7AH

TNNI1

C4orf48

CCK

PDZK1

MORN2

CCL19

NDUFB1

EIF4E1B

GTSF1

DCDC2

KLK7

CCPG1

LTB

LSP1

HES6

PIF1

NSUN4

CMC1

OTOP1

RPL29

B9D2

SPIN4

QPCT

NEUROD1

SCGB3A1

RF00017

TSPAN5

AKAP14

SYNPR

HTR1E

TNFSF13B

PPP1R1C

FRMPD1

ACTC1

PCYT1A

ANKRD9

.

C9H10orf120

TEX22

HTR2A

TMIE

CYP2A24

DNPH1

CTTNBP2NL

.

SCGN

CFC1

EFNB3

CSRP2

ZAP70

EFCAB11

GJD2

.

FGD3

EPB42

EFCC1

.

COX7C

GPR12

PEX10

KIF1B

GLI1

IGIP

COL22A1

WDR72

PF4V1

.

GPR101

CREB3L3

SLC6A7

SST

TGM5

DKK1

GPR183

PET100

MIS18A

CYP26A1

SAMD13

RAB38

FBXO15

SKA1

UBL5

CLEC1B

NR2E3

BMP4

MLPH

**Supplementary Data 6. The differentially expressed genes between female_mid and female_young macaques (female_mid lower than female_young, p value＜0.05)**

TMEM117

METTL13

PREX2

COL11A2

THBS1

ABCC4

UBE2W

ZIC4

CLASP1

ETNK1

CD46

DPP8

PGAP2

NAALAD2

TCF19

POLR1A

SLC46A3

FCRL6

ZNF33A

EGFR

COL1A2

RBPJ

NFATC3

LUM

NUFIP2

CD5L

TNS3

PHC3

ADD2

SMG1

TRIM5

UGGT1

MCOLN2

DOCK2

DAPP1

EPG5

ZFHX3

KMT2A

USP37

PER3

CYB561D1

REEP3

TNFRSF19

RIF1

PLA1A

HAVCR2

IL25

BDP1

CACNA1I

SCFD2

MRC1

MTNR1A

HMCN1

CLMN

DTX4

TRIM33

SLC4A5

ATP10B

PARP14

MKL2

CGNL1

OPHN1

SFN

CCDC150

NOS1

ABCC11

PLXDC2

PRKG1

CNTNAP5

RGS1

UNC13A

POLH

ELF4

SNX29

NAIP

ZCWPW2

ZBTB40

TLR7

MCC

EDEM3

HACD2

PLB1

ST8SIA5

COL21A1

KCNMB1

LRP6

VPS13D

HIPK1

MPEG1

ZNF354C

PCDH15

WWC2

SMC5

FNIP2

MAGI1

CDKL5

STOX2

PPM1K

LCP2

MYO9A

SSH1

BIRC5

EVC

NAV3

CD180

DNAAF4

N4BP2

TNKS

KCNJ5

NFIA

UNC5C

SYT10

LARP1

PLCG2

RFX3

NPFFR1

ZSCAN20

GRIK3

CCDC116

CRYBG1

TRIM34

PTGS1

RF01241

ZNF560

FRMPD4

ARFGEF3

UBN2

DENND5B

IGSF6

ZNF460

SPATA48

ELK4

GEN1

SP7

IGF2

NLRP1

VSIG4

ERC1

ATP2B4

STON2

FAM124B

MTTP

OTOA

ZNF844

SCN2B

RNGTT

SNX27

EFHB

LNPEP

ANKFY1

MFSD4B

LEPR

PSD3

MSR1

RCOR1

ALLC

AGO1

DMP1

TSPAN18

SH3RF3

SFMBT2

ZNF529

RF00105

BBX

ENPP4

SRGAP3

GIMAP8

SMURF1

PAK3

IRAK3

PNLDC1

SHISA9

C1orf87

KCNK13

ABL2

CCDC129

HMBOX1

ZFHX2

TANC2

PDYN

ATP8B4

SLC45A4

PTPN14

PARD3B

BMPR1B

ERCC4

RDM1

AMOTL1

CECR2

NT5C1A

C7

RF02153

RF00004

ADAMTS5

GAS7

CCR6

FOXN3

ARHGAP26

HMCN2

SP8

CDYL2

NBEAL1

SLCO5A1

WFDC1

SCEL

ZNF229

MTX3

MYO5C

NFATC2

AQP2

RF00017

TLR8

CACNG6

RECK

**Supplementary Data 7. The differentially expressed genes between male_mid and male_young macaques (male_mid higher than male_young, p value＜0.05)**

NFIB

SLC24A3

ALDH3A2

EYA1

UBL5

PPIL6

HMCN1

CCBE1

PYGL

MRPL39

MESP2

FSIP2

FCRL5

PTPRO

ECEL1

CRYBB2

GJB6

TMEM100

MIR6506

PNMA6A

CRYGN

NUP62

SORBS1

LAMB4

GLT8D2

HPGDS

MLLT3

ANGPTL1

SLC7A13

UBR1

TTR

HSD11B2

FGGY

TRIQK

SLC15A2

PTK2B

POPDC2

MRC1

CCNC

CD5L

COX17

HNRNPH2

DYRK4

SDC4

C21orf62

SORCS1

SLC39A12

CTTNBP2NL

CRABP1

AKAP3

TMEM72

FNDC1

C6H5orf49

IGF2BP1

C1QTNF6

NDUFA1

.

TUBA4B

SLC26A7

SLC14A1

SCUBE3

NR0B1

PHYHD1

RF01241

UPK3B

CLDN10

UBB

ZNF33A

TEX22

AQP6

FERMT1

MANSC4

LOR

ST8SIA4

SLC6A11

ECT2

CHRDL1

PDC

HNF4G

RF02153

RDH8

TNNT1

RAD51B

FAM13C

CD300E

CCDC187

RF00322

EHF

SCGN

LCN9

KCNJ5

ARMC4

ADRB3

UBQLNL

SMR3B

GPX5

.

CCDC129

LILRB4

CUBN

NOV

PLEKHN1

USH2A

ICOS

CDC42BPG

**Supplementary Data 8. The differentially expressed genes between male_mid and male_young macaques (male_mid lower than male_young, p value＜0.05)**

NXN

SPATA22

FAM50B

EMILIN2

HBB

TSPAN5

ZNF541

APOC1

CAV2

CRH

MYO1G

NUSAP1

SELENOW

CCL24

DPEP3

PBX4

PLEKHG4

L3MBTL4

PROSER2

OPRPN

FAM167B

OR2B11

OTOP1

ARHGAP11A

LMOD1

CSF2RB

ENC1

PTER

ST6GALNAC1

IZUMO2

FBP2

GCA

PSTPIP1

FCAR

FGD3

FAM160A1

BHLHE22

ADAD2

UCP2

FREM3

GPR143

CHRNA1

ZNF385B

FAM124B

EMC10

GZMM

.

RAMP3

LAT2

PVALB

ASPN

ANXA4

TENM4

CAVIN2

ZBTB42

GORASP1

TINAGL1

GPR101

BTNL3

IDI2

NLRC3

MMP9

BAIAP3

FAM196A

RFLNB

RHEBL1

ANO2

TNNI1

ZAP70

AQP2

RGS1

CCDC188

CD244

CENPF

CD93

MEDAG

SOCS3

UGT1A1

POLR2F

CFP

SNX31

CD70

SULT1B1

CTGF

GPR6

PCP2

CAPN3

TTF2

NCF4

KCNV2

SLC17A4

TMEM249

OXER1

.

NR2F2

SCIN

.

FAM170A

PKHD1

CD9

HGD

EPHB6

PRAM1

EGFLAM

RF00181

RHOH

GRIA1

ADIPOQ

CLEC7A

RF00599

CTSV

NEIL3

IRS4

TMC5

NGFR

CKM

TMEM30B

EDNRA

SCEL

WAS

CDH17

SAMD13

KCTD8

RF00560
